# Functional characterization of *Lilium lancifolium* cold-responsive Zinc Finger Homeodomain (*ZFHD*) gene in abscisic acid and osmotic stress tolerance

**DOI:** 10.7717/peerj.11508

**Published:** 2021-05-25

**Authors:** Yubing Yong, Yue Zhang, Yingmin Lyu

**Affiliations:** 1 Beijing Key Laboratory of Ornamental Plants Germplasm Innovation & Molecular Breeding, China National Engineering Research Center for Floriculture, Beijing Laboratory of Urban and Rural Ecological Environment, College of Landscape Architecture, Beijing Forestory University, Beijing, Haidian, China; 2 College of Landscape Architecture, Central South University of Forestry and Technology, Changsha, Hunan, China

**Keywords:** Zinc finger homeodomain (ZFHD) protein, Osmotic stress, Abscisic acid (ABA), Lily

## Abstract

**Background.:**

We have previously performed an analysis of the cold-responsive transcriptome in the mature leaves of tiger lily (*Lilium lancifolium*) by gene co-expression network identification. The results has revealed that a *ZFHD* gene, notated as encoding zinc finger homeodomain protein, may play an essential regulating role in tiger lily response to cold stress.

**Methods.:**

A further investigation of the *ZFHD* gene (termed as *LlZFHD4*) responding to osmotic stresses, including cold, salt, water stresses, and abscisic acid (ABA) was performed in this study. Based on the transcriptome sequences, the coding region and 5′ promoter region of *LlZFHD4* were cloned from mature tiger lily leaves. Stress response analysis was performed under continuous 4 °C, NaCl, PEG, and ABA treatments. Functional characterization of *LlZFHD4* was conducted in transgenic *Arabidopsis*, tobacco, and yeast.

**Results.:**

*LlZFHD4* encodes a nuclear-localized protein consisting of 180 amino acids. The N-terminal region of LlZFHD4 has transcriptional activation activity in yeast. The 4 °C, NaCl, PEG, and ABA treatments induced the expression of *LlZFHD4*. Several stress- or hormone-responsive cis-acting regulatory elements (T-Box, *Box*I. and ARF) and binding sites of transcription factors (MYC, DRE and W-box) were found in the core promoter region (789 bp) of *LlZFHD4*. Also, the *GUS* gene driven by *LlZFHD4* promoter was up-regulated by cold, NaCl, water stresses, and ABA in *Arabidopsis*. Overexpression of *LlZFHD4* improved cold and drought tolerance in transgenic *Arabidopsis*; higher survival rate and better osmotic adjustment capacity were observed in *LlZFHD4* transgenic plants compared to wild type (WT) plants under 4 °C and PEG conditions. However, *LlZFHD4* transgenic plants were less tolerant to salinity and more hypersensitive to ABA compared to WT plants. The transcript levels of stress- and ABA-responsive genes were much more up-regulated in *LlZFHD4* transgenic *Arabidopsis* than WT. These results indicate *LlZFHD4* is involved in ABA signaling pathway and plays a crucial role in regulating the response of tiger lily to cold, salt and water stresses.

## Introduction

Plants have evolved a series of adaptive responses to cope with environmental stresses, such as salinity, drought, and low temperature. Many of these adaptations occur at the molecular level: start with signal perception, move to signal relay, and end with gene expression. Transcription factors (TFs) play pivotal roles in this signaling transduction event, regulating the expression of multiple stress-inducible genes by specifically binding to the corresponding cis-acting elements (Khatun et al., 2017; Huang et al., 2009; Zang et al., 2017). Recently, a homeobox TF, zinc finger-homeodomain (ZFHD), has attracted attention for its functions on mediating plant developmental processes and abiotic stress responses, which can bind to a core consensus sequence of ATTA and form homodimers and heterodimers (Sun et al., 2021).

The ZFHD TFs contain two highly conserved domains: the N-terminal C2H2-type zinc finger (ZF) domain and the C-terminal homeodomain (HD) domain (Wang et al., 2014). The ZF, one of the most critical structural motifs, widely exists in many regulatory proteins. The ZF consists of a single zinc ion in the core surrounded by two pairs of conserved cysteine (Cys) and, or histidine (His) residues, which participates in DNA binding and protein-protein interactions (Krishna, Majumdar & Grishin, 2003; Takatsuji, 1999). According to the presence of Cys and His residues, ZFs are classified into different types, such as Cys3His (C3H), Cys2His2 (C2H2), and Cys2Cys2 (C2C2) (Chen et al., 2020; Englbrecht, Schoof & Böhm, 2004; Halbach, Scheer & Werr, 2000; Kosarev, Mayer & Hardtke, 2002; Zang et al., 2017). Among them, C2H2-type ZFs act as essential regulators in many plant stress responses or other metabolic pathways (Xie et al., 2019; Zang et al., 2017). The HD, as a well-characterized DNA-binding domain (BD), containing a conserved 60-amino acid motif, is present in TFs in all eukaryotic organisms (Hu, De Pamphilis & Ma, 2008; Mukherjee, Brocchieri & Bürglin, 2009). Most HD-containing proteins are related to additional domains or motifs for various regulatory functions, can also be divided into different subgroups, including ZFHD, leucine zipper-associated HD (HD-ZIP) and Knotted-related homeobox (KNOX) proteins, etc. (Ariel et al., 2007).

The *ZFHD* gene family was first found in *Flaveria trinervia* (Windhövel et al., 2001), and was subsequently identified in many other plant species by genome-wide study (Sun et al., 2021; Liu, Yang & Zhang, 2021). Meanwhile, there is increasing evidence indicating that *ZFHD* genes play vital roles in plant stress response (Khatun et al., 2017; Shalmani et al., 2019; Wang et al., 2016). For example, drought, salt, and cold stresses up-regulated the expression of *AtZFHD04* in *Arabidopsis thaliana* (Barth et al., 2009). *AtZFHD1* was induced by drought and salt treatments. Co-overexpression of both the *AtZFHD1* and *AtNAC* gene could activate the expression of dehydration1 (*ERD1*) and other stress-inducible genes, improve the drought tolerance in transgenic *Arabidopsis* (Tran et al., 2007). AtZFHD10 was found to interact with TANDEM ZINC-FINGER PLUS3 (TZP) to modulate hormone signaling in stress response (Perrella et al., 2018). Four *OsZFHD* genes in rice (*Oryza sativa*) were shown to be involved in cold and drought stress responses, which can bind to the promoter region of *OsDREB1B* (Figueiredo et al., 2012). In Chinese cabbage (*Brassica rapa* ssp. *pekinensis*), over half of 31 *ZFHD* genes were up-regulated after heat, cold, and salt treatments but down-regulated after drought (Wang et al., 2016). In tomato (*Solanum lycopersicum*), the transcript level of *SlZFHD19* and *SlZFHD20* markedly increased after drought, heat, and cold stress treatments; *SlZHD2*, *SlZHD7*, *SlZHD8*, and *SlZHD15* were up-regulated under drought and salt treatments (Khatun et al., 2017). More recent studies showed that four *CsZFHD*s in cucumber (*Cucumis sativus*) were significantly down-regulated by drought stress (Lai et al., 2021). In wheat (*Triticum aestivum*), ten *TaZFHD* genes were mainly up-regulated under salt, cold and water stress treatments (Liu, Yang & Zhang, 2021). *NtZFHD21* from tobacco (*Nicotiana tabacum*) was highly expressed in response to the drought treatment (Sun et al., 2021).

On the other hand, the phytohormone, abscisic acid (ABA), has been reported to play a predominant role in regulating plant response to multiple environmental stimuli (Chong, Guo & Zhu, 2020); thus, the ABA signaling pathway has been studied extensively (Chen et al., 2020; Dar et al., 2017). ABA can trigger extensive changes in the transcriptome to help plants adapt to abiotic stresses (Verma, Ravindran & Kumar, 2016). So far, many TF genes from ABA-responsive element binding factors (ABF), MYC, MYB and NAC families have been identified as functioning in the transcriptional regulation of ABA-mediated stress-inducible expression. Also, some *ZFHD* genes were induced by ABA treatment, such as *AtZFHD1 OsZHD4*, *BraZF-HD03* and *BraZF-HD05* (Figueiredo et al., 2012; Tran et al., 2007; Wang et al., 2016). However, the involvement of *ZFHD* genes in the ABA-dependent or -independent signaling pathway is still largely unknown.

**Figure 1:**
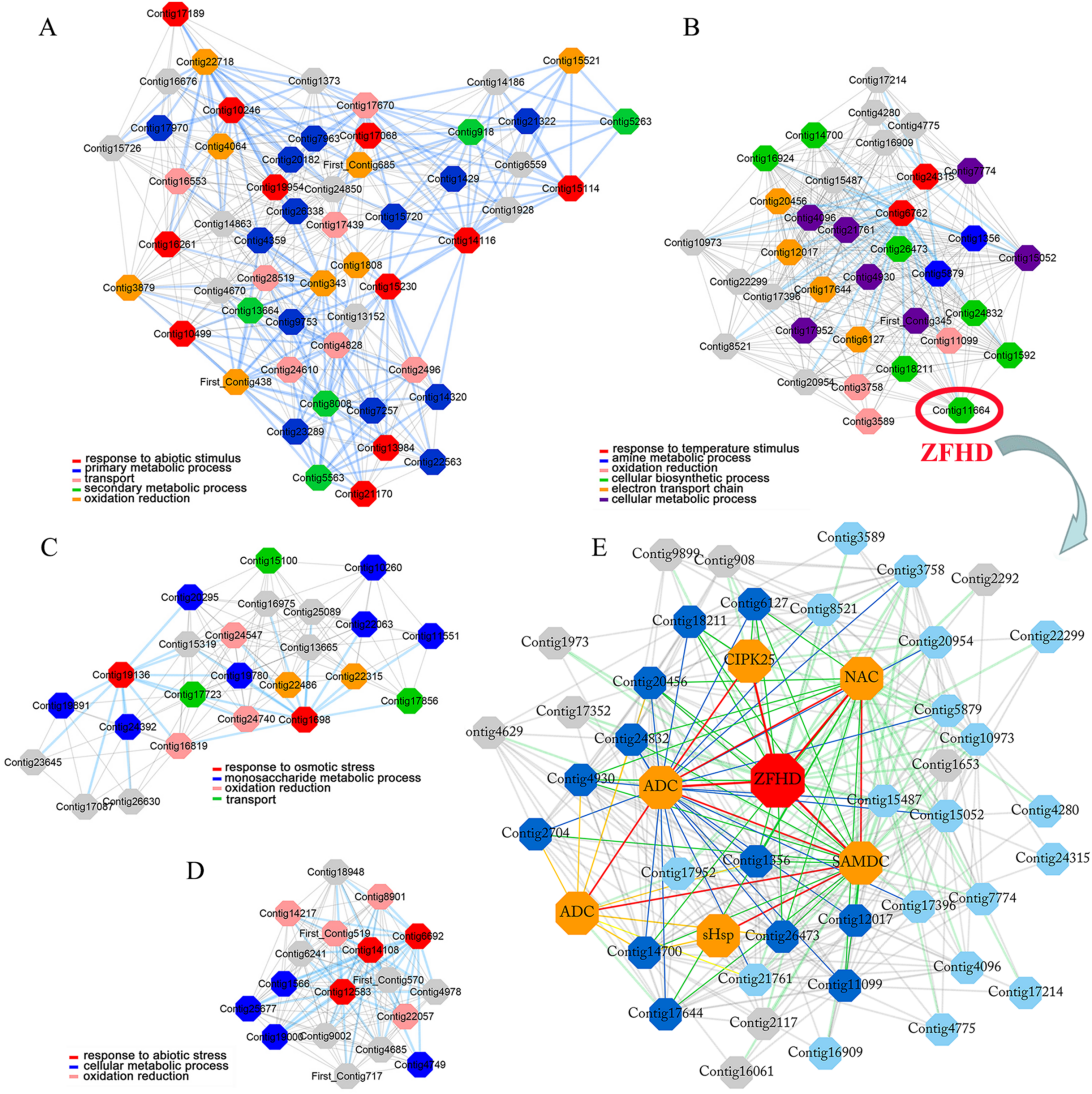
Four stress response enriched gene modules (A, B, C, D) and *LlZFHD4* co-expressed gene network (E) in tiger lily (*L. lancifolium*) response to cold stress.

Lily is one of the most important flower crops in the world, and it is susceptible to low temperature, high salinity and drought stresses. However, as the most widely distributed wild lily in East Asia, tiger lily (*Lilium lancifolium*) has been reported to have distinctive molecular mechanisms that confer its superior tolerance to various abiotic stresses (Wang et al., 2014). Based on the cold-responsive (4 °C-treated 0, 2, and 16 h) transcriptome in the mature leaves of tiger lily, we have constructed the highest reciprocal rank-based gene co-expression network in our previous study (Yong et al., 2018). Four gene modules were identified due to their GO terms significantly enriched in stress response (Figs. 1A, 1B, 1C, 1D) (Yong et al., 2018). A *ZFHD* gene was found to be a hub gene in one of these four modules (Fig. 1B). This *ZFHD* gene co-expressed with some known stress-related genes, including *CIPK25* (CBL-interacting protein kinase), *ADC* (Arginine decarboxylase), *SAMDC* (S-adenosylmethionine dehydrogenase), and another TF gene from NAC family (Fig. 1E) (Yong et al., 2018). In this study, we cloned and analyzed this *ZFHD* gene *LlZFHD4*. The subcellular localization and transcription activation of LlZFHD4 protein were observed. The functions of *LlZFHD4* in response to osmotic stress and ABA in tiger lily were detected and discussed further.

## Materials & methods

### Plant materials

The bulbs of wild tiger lily (*Lilium lancifolium*) with a size of 3–5 cm collected from Heilongjiang province, China, were used in this study. The bulbs were washed with tap water, sterilized with carbendazim, air-dried, covered with a 1-cm layer of moist vermiculite, and stored at 4 °C for 40 days. After vernalization, the bulbs were cultured in the science and technology experimental greenhouse of Beijing Forestry University (116.3° E, 40.0° N).

*Arabidopsis thaliana* ecotype Columbia-0 (Col-0) was used for the ectopic expression study; tobacco *Nicotiana benthamiana* (NT78) was used for the subcellular localization study. The seeds of tobacco and *Arabidopsis* were sown in plastic containers filled with the sterile substrate (peat:vermiculite = 1:1), and cultured in a growth chamber with a light/dark regime 16/8 h (100 µmol·m^−2^·s^−1^ ), at 22/16 °C, and 65% relative humidity. The containers were covered with plastic wrap under growth chamber condition until seed germination.

### Gene cloning and sequence analysis

RNAisomate RNA Easyspin isolation system (Aidlab Biotech, China) was used for total RNA extraction. The RNA quantity was examined by NanoDrop 2000 (Thermo Scientific, Waltham, MA, USA). The First-strand synthesis of cDNA was produced using PC54-TRUEscript RT kit (+gDNA Erase) (Aidlab Biotech, China) using 1 µg total RNA.

The complete cDNA sequence of *LlZFHD4* was obtained from tiger lily’s RNA-seq data published before (Yong et al., 2018; Yong, Zhang & Lyu, 2019b). Open reading frame (ORF) of *LlZFHD4* was amplified using PC09-2×Taq PCR MasterMix (Aidlab Biotech, China) with the forward primer 5′-ATGGATCTCCCCATTTATC-3′ and the reverse primer 5′-TCAGTAGTCACTCTGCAATG-3′. The PCR reaction system and procedure were performed according to the manufacturer’s protocol. The PCR products were ligated into the pEASYT1-Blunt cloning vector (TransGen Biotech, Beijing, China) and sequenced by the Beijing TransGen Biotech Company.

Amino acid sequence alignment analyses were performed by BLASTN in the National Center for Biotechnology Information (NCBI) and DNAMAN (version 7). The unrooted phylogenetic trees were constructed in MEGA5 software using the neighbor-joining method with 1,000 replications. The online database ExPASy (http://expasy.org/tools/protparam.html) was used for the theoretical molecular weight and isoelectric point calculation.

### Promoter cloning and sequence analysis

DNeasy Plant Mini Kit (Qiagen, Valencia, CA, USA) and Genome Walker Kit (Clontech, CA, USA) were used for genomic DNA extraction from mature leaves of tiger lily and *LlZFHD4* promoter cloning, respectively (Yong, Zhang & Lyu, 2019a, 2019b). The promoter region of *LlZFHD4* was amplified by nested PCR methods according to the user manual of Genome Walker Kit (Clontech, CA, USA) with the primary PCR primer GSP1: GGGTGGCTGCTAGGGTCAAACAGTAAT and the secondary PCR primer GSP2: AAGGTGGTTACAATGGGAAGAAGATG. Using touchdown PCR method, the primary PCR reaction procedure was as follows: pre-denaturation at 94 °C for 1 min; seven cycles of denaturation at 94 °C for 25 s, 65–59 °C for 30 s (decrease 1 °C per cycle), 72 °C for 3 min; 32 cycles of denaturation at 94 °C for 25 s, 57 °C for 30 s, 72 °C for 30 s; and extension at 72 °C for 10 min. The 1/50 dilution of the primary PCR product was used as a template in the secondary PCR. The reaction procedure was as follows: pre-denaturation at 94 °C for 5 min, 35 cycles of denaturation at 94 °C for 40 s, 58 °C for 40 s, 72 °C for 3 min, and extension at 72 °C for 30 s. The prediction of conserved cis-element motifs was performed on PLACE (http://www.dna.affrc.go.jp/PLACE/signalscan.html) databases.

### Quantitative real-time PCR (qRT-PCR) analysis

Primer Premier 5.0 was used for primer design: primer annealing temperature 50–65 °C, primer length 18–24 bp, and amplification length 80–250 bp. The primers of *LlZFHD4* and *AtRD29A*, *AtRD29B*, *AtRD20*, *AtLEA14*, *AtGolS1*, *AtAPX2* from *Arabidopsis* were listed in Table S1. The SYBR^®^ qPCR mix (Takara, Dalian, China) and Bio-Rad/CFX Connect^™^ Real-Time PCR Detection System (Bio-Rad, Irvine, CA, USA) were used in the qRT-PCR experiment. The reaction procedure was as follows: pre-denaturation at 95 °C for 30 s, 40 cycles of denaturation at 95 °C for 5 s, annealing temperature for 30 s, and extension at 72 °C for 30 s. Relative mRNA content was calculated using the 2^−ΔΔCt^ method. Each sample was amplified in biological and technical triplicate. Tiger lily *LlTIP1* (Wang et al., 2014) and *Arabidopsis Atactin* (NM_112764) were used as internal reference genes (Table S1).

### Subcellular distribution of LlZFHD4

The ORF of *LlZFHD4* without stop codon was amplified with the forward primer 5′-CATTTACGAACGATACTCGAG(*Xho*I)ATGGATCTCCCCATTTATC-3′ and the reverse primer 5′-CACCATCACTAGTACGTCGAC(*Sal*I)GTAGTCACTCTGCAATG-3′. The amplified product was inserted into the *Xho*I and *Sal*I site of the pBI121-GFP vector driven by a CaMV 35S promoter according to the ClonExpress II kit’s user manual (Vazyme, Nanjing, China). The confirmed recombinant vector pBI121-LlZFHD4-GFP was transformed into *Agrobacterium* strain GV3101 competent cells by the freeze-thaw method; and introduced in the tobacco leaf epidermal cells by the infiltration method with GV3101 cells. After 32 h incubation, the GFP fluorescence signals were detected in agroinfiltrated tobacco leaves using Leica TCS SP8 Confocal Laser Scanning Platform (Yong, Zhang & Lyu, 2019a, 2019b).

### Yeast transcription activation assay of LlZFHD4

Using a ClonExpress II kit, the entire coding region (1–540 bp) and N-terminus (1–270 bp) and C-terminus (271–540 bp) of the *LlZFHD4* cDNA region were amplified (primers are shown in Table S1), and cloned into the *EcoR*I and *BamH*I site of pGBKT7 vector MCS region (Clontech, CA, USA), resulting in recombinant vector pGBKT7-LlZFHD4-A (1–180 aa), pGBKT7-LlZFHD4-N (1–90 aa) and pGBKT7-LlZFHD4-C (91–180 aa). The confirmed recombinant vectors were introduced into the Y2HGold yeast strain (Huayueyang, Beijing, China) according to our previous study (Yong, Zhang & Lyu, 2019a, 2019b). The 1/10 and 1/100 dilution transformed yeast cells were incubated on plates containing the appropriate SD selection medium (Cao et al., 2017) at 30 °C. Yeast colony formation was photographed after three days.

### Generation of *LlZFHD4* transgenic *Arabidopsis*

The entire coding region of *LlZFHD4* was inserted into the pBI121 vector driven by CaMV 35S for ectopic expression study. The CaMV35S promoter region in the pBI121-GUS vector was replaced by the *LlZFHD4* promoter region for promoter activity analysis. After being mobilized to *Agrobacterium* strain GV3101, the recombinant vectors were transformed into wide-type (WT) *Arabidopsis* with GV3101 cells through the floral dip method. The MS medium containing 50 mg/L kanamycin was used to screen the progeny transgenic plant seeds. T3-generation of *LlZFHD4* transgenic lines were harvested after qRT-PCR confirmation; T2-generation of *LlZFHD4* promoter transgenic lines were collected after PCR confirmation with promoter-specific primers: forward primer 5′-GCTTGATATCGAATTCG-3′ and reverse primer 5′-TCTCAATTTAGGATCCT-3′.

### Stress treatments

**Figure 2:**
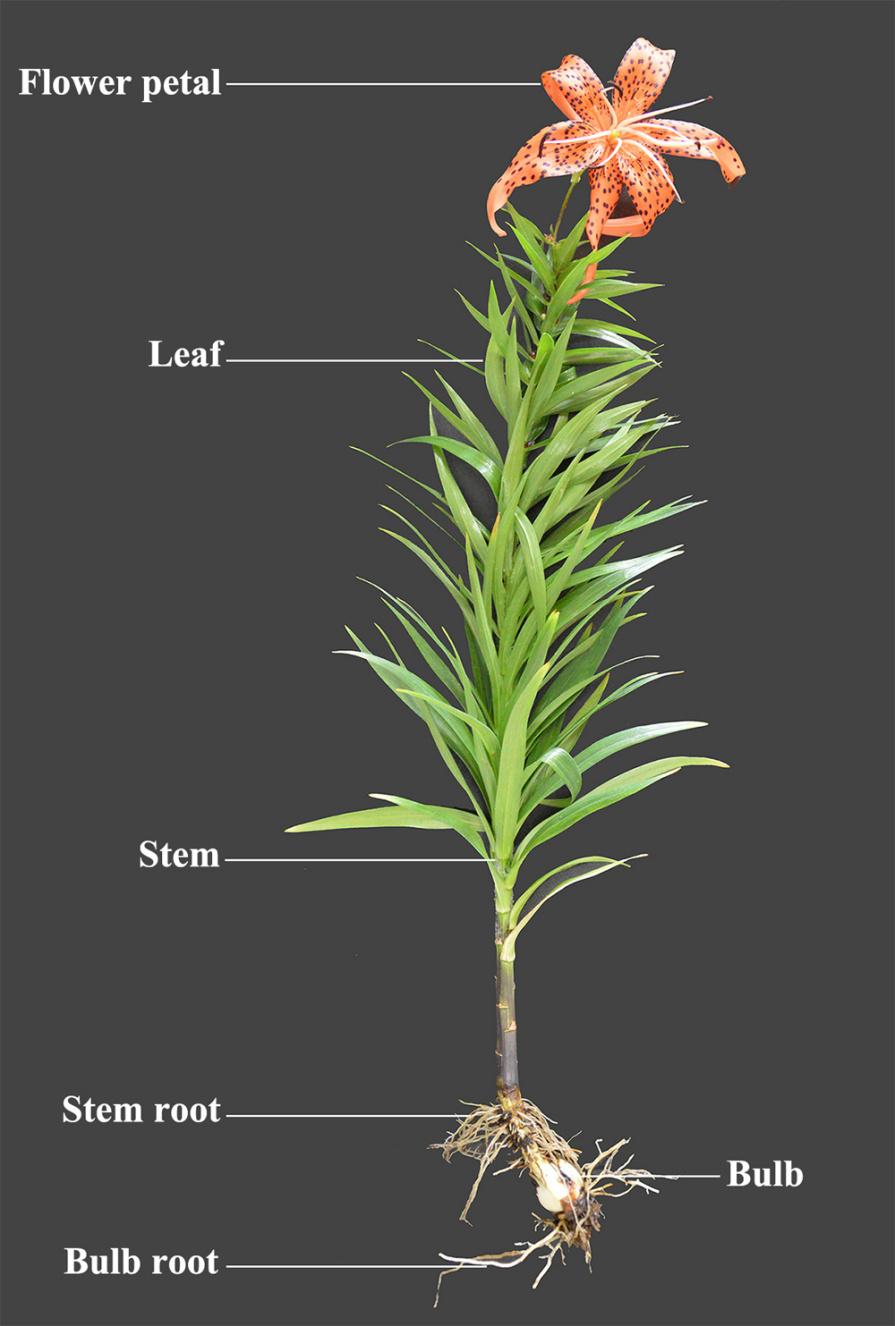
The collection samples of tiger lily (*L. lancifolium*).

Seedlings during the blooming period were used for stress experiments (Fig. 2). Mature leaves, bulbs, bulb roots, stem roots, stems, and flower petals were sampled, respectively. All tissues and organs were directly frozen in liquid nitrogen and stored at −80 °C for gene expression analysis.

Cold, dehydration, salinity, and ABA treatments were performed on tiger lily by treating eight-week-old seedlings with 4 °C, 16.1 % PEG6000 (−0.5 MPa), 100mM NaCl, and 100µM exogenous ABA for 0, 1, 3, 6, 12, 24 h before sampling for expression analysis of *LlZFHD4* (Yong, Zhang & Lyu, 2019a, 2019b). Similarly, three-week-old *LlZFHD4* promoter transformant *Arabidopsis* plants were also treated with the above conditions for *GUS* gene expression assay. Three-week-old *LlZFHD4* transgenic *Arabidopsis* and WT seedlings were harvested under normal conditions for expression analysis of *AtRD29A*, *AtRD29B*, *AtRD20*, *AtLEA14*, *AtGolS1*, and *AtAPX2* genes (Table S1). All experiments were repeated three times in biological triplicates.

### Stress tolerance assays in *LlZFHD4* transgenic *Arabidopsis*

Three-week-old T3-generation transgenic *Arabidopsis* lines and WT seedlings were pretreated under 4 °C for 3 h, and then treated under –4, –6, or –8 °C for 12 h as cold treatment (Yong, Zhang & Lyu, 2019a, 2019b). The water intake of seedlings was withheld for 30 days as drought treatment (Yong, Zhang & Lyu, 2019b). After stress treatments, the stress-treated *Arabidopsis* seedlings were recovered in the growth chamber under normal growth conditions for seven days. The survival rate of *Arabidopsis* seedlings was scored. The relative electrolyte leakage and soluble sugar content were determined before (control) and after 3 h 4 °C and 16.1% PEG6000 (−0.5 MPa) treatments by the thermal conductivity measurement method and the anthrone method, according to Zhang et al. (2015). The measuring method of water loss rate was described in previous study of Cao et al. (2007).

Seeds of selected T3-generation homozygous transgenic lines and WT were sowed on MS medium containing 2 µM ABA or 50 mM NaCl for seven days (Yong, Zhang & Lyu, 2019a); and then the germination was scored and photographed.

## Results

### Gene isolation and sequence analysis of *LlZFHD4*

**Figure 3:**
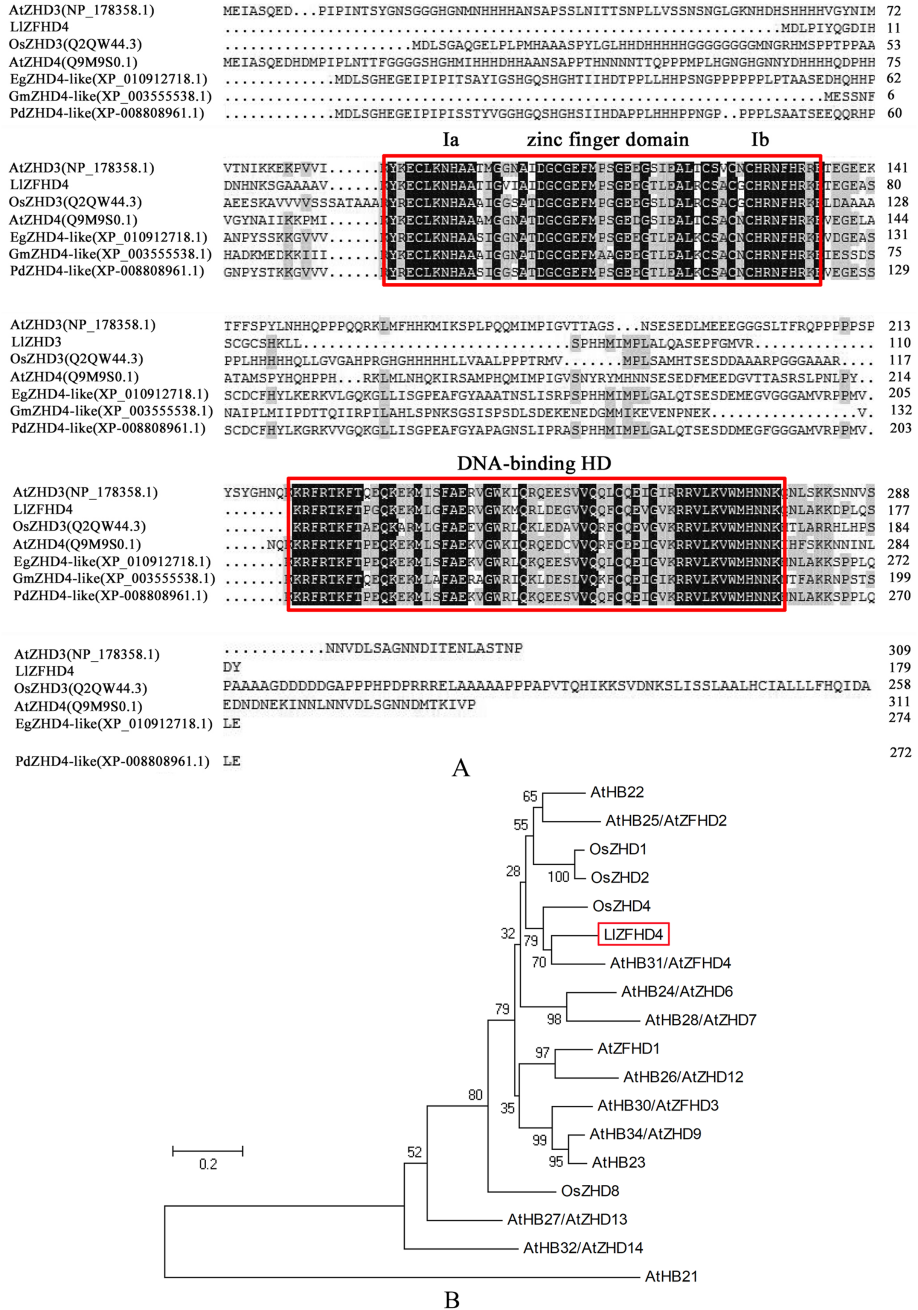
Conserved domain and phylogenetic analysis of LlZFHD4 protein. (A) Alignment of LlZFHD4 with *Arabidopsis* AtZHD3 and AtZHD4, rice OsZHD3, *Elaeis guineensis* EgZHD4-like, *Glycine max* GmZHD4-like and *Phoenix dactylifera* PdZHD4. (B) Phylogenetic tree analysis of LlZFHD4 with *Arabidopsis* AtZHD3 (NP_178358.1), AtZHD4 (Q9M9S0.1), AtZFHD2 (NP_201344.1), AtZFHD1 (NP_177118.1), AtZHD14 (NP_563956.1), AtHB21 (OAP10319.1), AtZHD6 (NP_565436), AtZHD9 (NP_189534), AtZHD7 (NP_190658), AtHB22 (NP_850266.1), AtZFHD3 (NP_197025.1), AtHB23 (OAP18806.1), AtZHD13 (NP_199092.1), AtZHD12 (NP_200856.2), AtZFHD2 (NP_201344.1), and rice OsZHD1 (Q6YXH5.1), OsZHD2 (Q6ZB90.1), OsZHD4 (Q53N87.2), OsZHD8 (Q7X7N3.1) proteins.

*LlZFHD4* gene comprises 543 bp open reading frame (ORF) corresponding to a protein of 180 amino acids with a calculated molecular mass of 20.08 kDa and a pI of 8.91 (Fig. S1). The LlZFHD4 contained a DNA-binding homeodomain in the C-terminus, and a conserved zinc finger domain in the N-terminus; two segments Ia and Ib were located in the zinc finger domain (Fig. 3A). Amino acid sequence alignment results showed that LlZFHD4 shared 67%, 47%, 60%, 59%, 54%, 53% identities with AtZHD4 and AtZHD3 from *Arabidopsis*, PdZHD4 from *Phoenix dactylifera*, EgZHD4-like from *Elaeis guineensis*, OsZHD3 from rice, GmZHD4-like from *Glycine max* (Fig. 3A). A phylogenetic tree was constructed based on the known amino acid sequences of *ZFHD* genes in the model plant (*Arabidopsis*) and crop (rice), showing that the *LlZFHD4* sequence clustered closely with *Arabidopsis* AtZFHD4 and rice OsZHD4 shared 67% and 55% identities, respectively (Fig. 3B).

### LlZFHD4 is a nucleus-localized transcriptional activator

**Figure 4:**
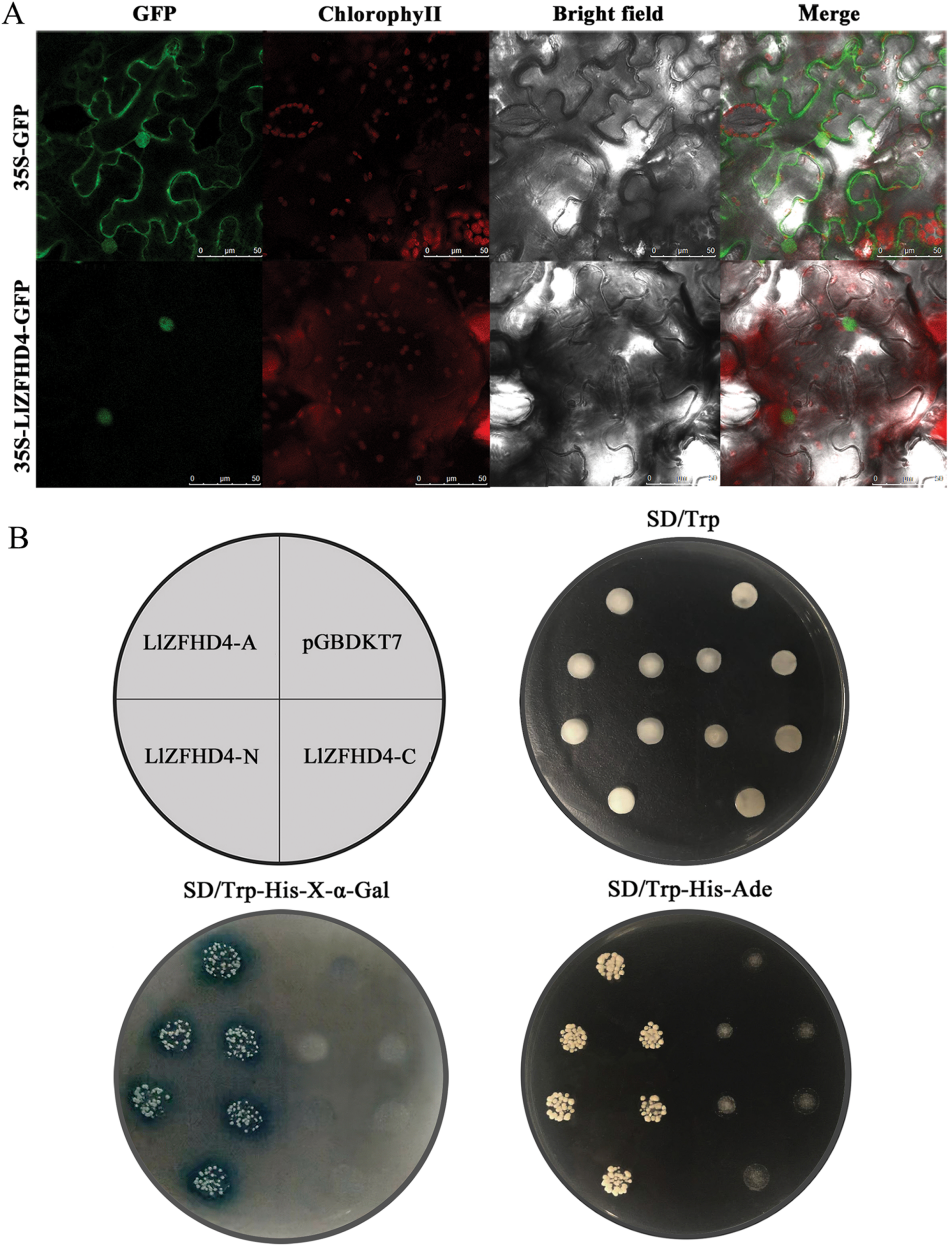
Subcellular localization and transactivation assay of LlZFHD4. (A) Subcellular localization of free GFP and LlZFHD4-GFP under the control of CaMV 35S promoter by transient expression assay in tobacco leaf epidermal cells (scale bars=50 μm). Green fluorescence represents GFP fluorescence. ChlorophyII auto-fluorescence is in red. Bright-field images show the equivalent field observed under white light. All of the signals were monitored by confocal microscopy. (B) Recombinant pGBKT7 vector LlZFHD4-A (entire coding region), LlZFHD4-N (N-terminal region), LlZFHD4-C (C-terminal region) and empty pGBDKT7 vector (control) were introduced into the Y2HGold yeast strain. The 1/100 dilution transformed yeast cells were incubated on plates containing the appropriate SD selection medium.

The *LlZFHD4* ORF without stop codon was connected with the N-terminal of the *GFP* gene driven by a CaMV 35S promoter. The 35S-LlZFHD4-GFP fusion protein was observed in the subcellular localization of LlZFHD4. A nuclear localization signal was detected in 35S-LlZFHD4-GFP transformed tobacco leaf epidermal cell; in contrast, a ubiquitous distribution signal in the whole cell was detected in control (35S-GFP) (Fig. 4A), suggesting LlZFHD4 is a nucleus-localized protein.

To investigate whether the LlZFHD4 has transactivation activity, recombinant pGBKT7 vector LlZFHD4-A (entire coding region), LlZFHD4-N (N-terminal region), LlZFHD4-C (C-terminal region), and pGBDKT7 vector (control) were introduced into the Y2HGold yeast strain. The LlZFHD4-A and LlZFHD4-N contained yeast strain grew well on the SD/-Trp/-His/-Ade medium, and the colonies are blue on SD/-Trp/-His-x-α-gal medium. These results indicate the LlZFHD4 has transcriptional activity, and the deletion of the C-terminal region (from the position of 91 to 180 aa) did not affect the activation (Fig. 4B), which means that LlZFHD4 is a transcription activator with transactivation in the N-terminus.

### Expression patterns of *LlZFHD4* under stresses

**Figure 5:**
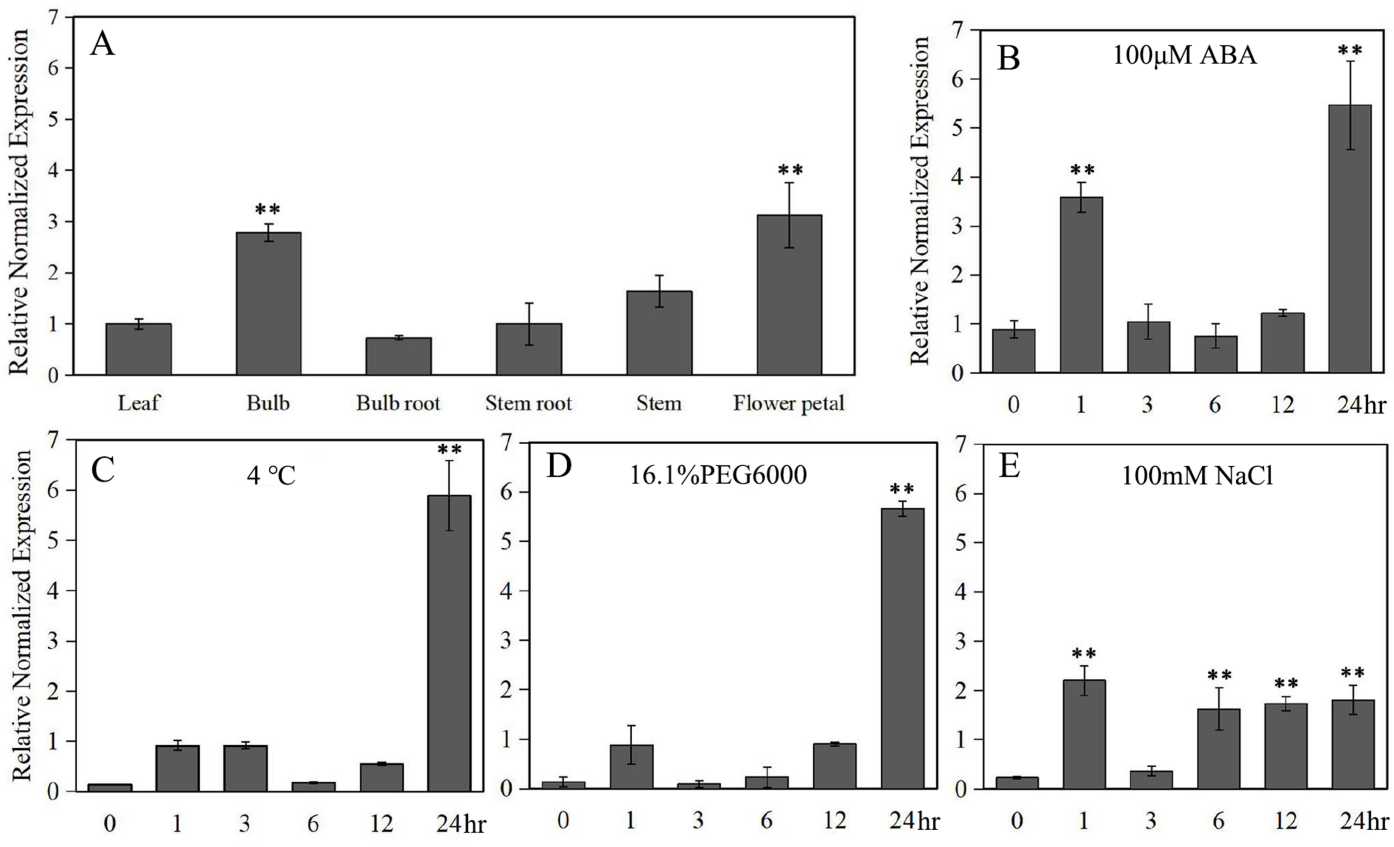
Quantitative real-time analysis of *LlZFHD4*. The transcript level of *LlZFHD4* in (A) mature leaf, bulb, bulb root, stem root, stem and flower petal under normal condition, and in leaf after (B) ABA, (C) cold, (D) drought and (E) salt treatments. Columns show relative expression levels of *LlZFHD4* normalized against levels of *LlTIP1* as calculated by qRT-PCR (mean ± SD of three biological replicates). Significant difference **p-value < 0.01.

Under normal conditions, *LlZFHD4* was expressed in all detected organs of tiger lily, including mature leaf, bulb, bulb root, stem root, stem, and flower petal. The transcript level of *LlZFHD4* was shown to be the highest in flower petal followed by bulb, while it was low in leaf and stem (Fig. 5A). Under ABA (100 μM) treatment, the expression of *LlZFHD4* was highly induced within 2 h with a threefold to fourfold increase, and then peaked at 24 h (Fig. 5B). Similarly, salt (NaCl 100 mM) treatment also induced the expression of *LlZFHD4* within 2 h showing a twofold to threefold increase (Fig. 5E). However, treatment of plants with cold (4 °C) and drought (16.1% PEG6000) stresses could not up-regulate the expression of *LlZFHD4* until 24 h with a fivefold to sixfold increase (Figs. 5C, 5D). These results showed that *LlZFHD4* is cold, drought, and salt-responsive (Table S2 lists all the raw data of Fig. 5).

### Promoter analysis of *LlZFHD4* in response to stresses

**Figure 6:**
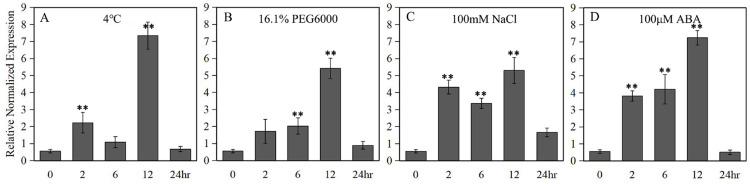
*GUS* transcript levels in *LlZFHD4* promoter-transformed *Arabidopsis* under (A) cold (4 °C), (B) drought (16.1% PEG6000), (C) salt (NaCl 100 mM) and (D) ABA (100 μM). Columns indicate relative expression levels of *GUS* normalized against levels of *Atactin* as calculated by qRT-PCR (mean ±SD of three biological replicates). Significant differences between the stress-treated and untreated *LlZFHD4* promoter-transformed plants are indicated as **p-value < 0.01.

A 789 bp upstream of the ATG start codon of the *LlZFHD4* gene was cloned and used as the *LlZFHD4* promoter sequence (Fig. S2). Putative cis-acting regulatory elements annotated as stress- or hormone-responsive elements (T-Box, *Box*I. and ARF elements) were identified (Table 1). Three binding sites (MYC, DRE, and W-box) of MYC, DREB, and WRKY TFs were also found in the promoter region of *LlZFHD4*. The expression of the *GUS* gene driven by the *LlZFHD4* promoter was detected by qRT-PCR in transgenic *Arabidopsis* seedlings. The qRT-PCR results showed that treatment of transgenic *Arabidopsis* with cold (4 °C), drought (16.1% PEG6000), salt (NaCl 100 mM), and ABA (100 μM) could induce *GUS* gene with a maximal transcript level at 12 h, leading to a five fold to eightfold increase (Fig. 6); suggesting the promoter activity of *LlZFHD4* can be induced by these stresses in some degree (Table S3 lists all the raw data of Fig. 6).

**Table 1:** Putative cis-acting elements identified in the promoter region of *LlZFHD4*.

Site name	(Strand) position	Sequence	Function
MYCRS	(+)145,502; (−)46	CAA(A/G)TG CAGCTC	MYC recognition site involved in cold and drought-inducibility
*Box*I	(−)318	TTTCAAA	light responsive element
T-Box	(−)307	ACTTTG	light responsive element
SORLIP	(+)55	GGGCC	Sequences Over-Represented in Light-Induced Promoters (SORLIPs)
G-BOX	(−)399	CACGTC	cis-acting regulatory element involved in light responsiveness
ARE	(+)234	TGGTTT	cis-acting regulatory element essential for the anaerobic induction
DRE	(+)759; (−)458	GTCGAC ACCGAC	dehydration-responsive element (DRE)
ARF	(−)535	TGTCTC	ARF (auxin response factor) binding site
GARE-motif	(+)238	TCTGTTG	Gibberellin-responsive element
W-box	(+)328; (−)305	TTGAC(C/T)	WRKY proteins bind specifically to the DNA sequence motif (T)(T)TGAC(C/T)

### Overexpressing *LlZFHD4* alters the abiotic stress tolerance of transgenic Arabidopsis

**Figure 7:**
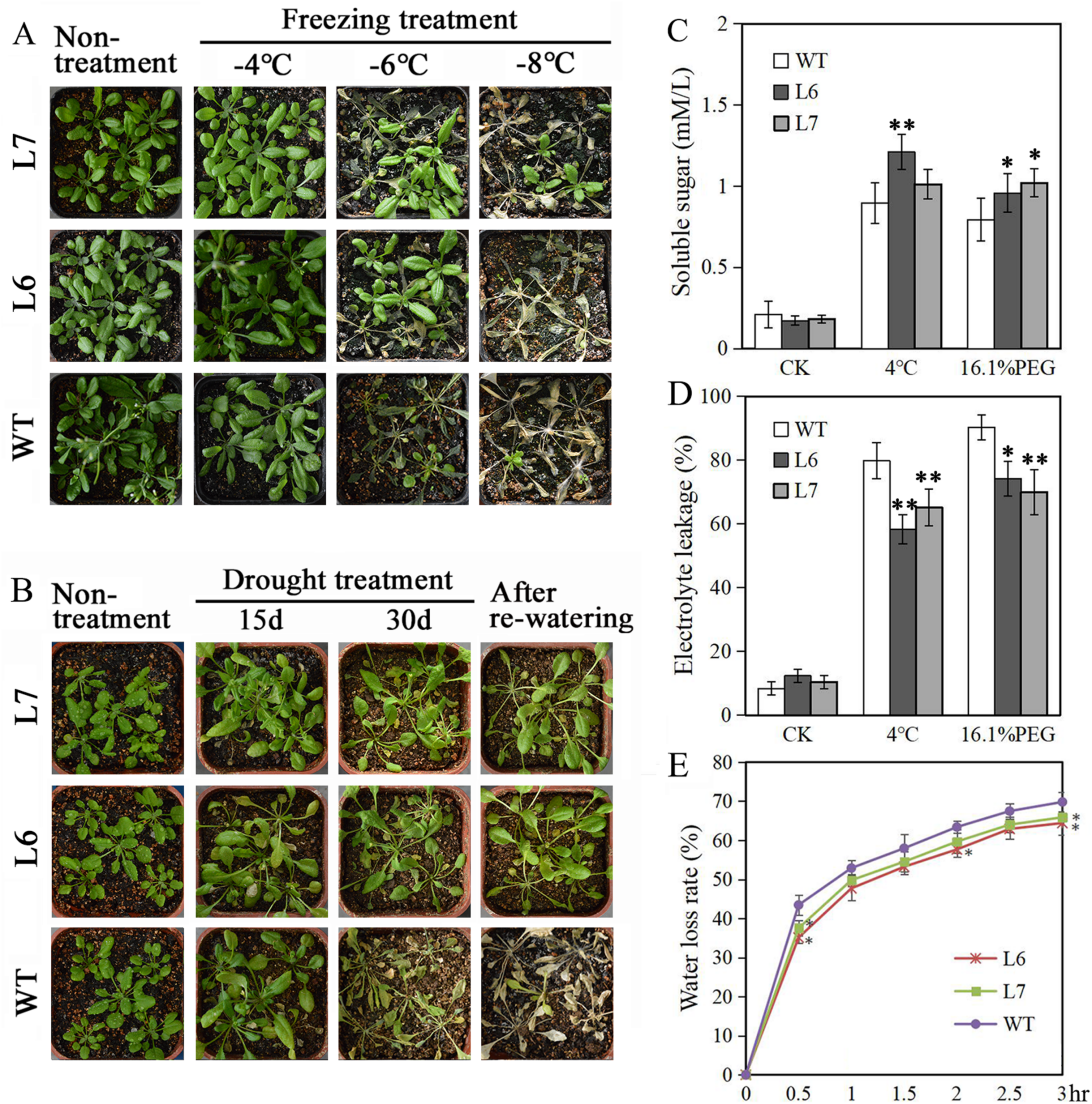
Overexpressing *LlZFHD4* in *Arabidopsis* enhanced cold and water stress tolerance. Observation of T3 generation *LlZFHD4* transgenic Arabidopsis lines (L6, L7) and WT seedlings after (A) freezing and (B) drought treatments. (C) Relative electrolyte leakage and (D) soluble sugar content in L6, L7 and WT plants after 4 °C and 16.1% PEG treatments. (E) The rate of water-loss from L6, L7 and WT cutting leaves. Each experiment comprises 30 plants. The bars show the mean ± SD of three biological replicates. Significant differences between the L6, L7 and WT plants are indicated as 0.01 < *p-value < 0.05 and **p-value < 0.01.

Two T2 generations *LlZFHD4* transgenic *Arabidopsis* lines, Line 6 and Line 7 (L6, L7), with relatively high LlZFHD4 transcript levels, were chosen by qRT-PCR (Fig. S3). The T3 generation of L6 and L7 were used for subsequence stress tolerance analysis. Under normal growth conditions, L6, L7, and WT *Arabidopsis* seedlings all grew well. No difference in plant morphology between L6, L7, and WT plants was noticed (Figs. 7A, 7B). However, under cold and water stress treatments, the less damaging effects were observed on L6 and L7, compared to WT. After exposing to freezing temperatures (especially under −6 °C) for 12 h or withholding water for 30 days, L6 and L7 seedlings displayed better growth status with larger leaf area, and significantly higher survival rate as compared to WT plants (Figs. 7A, 7B). Additionally, some essential physiological parameters, including water-loss rate, relative electrolyte leakage, and soluble sugar content, were measured before (control) and after 3 h 4 °C and 16.1% PEG6000 (−0.5 MPa) treatments. We found that L6 and L7 plants showed lower water-loss rates and electrolyte leakage amounts, higher soluble sugar levels than WT plants (Figs. 7C, 7D, 7E). These results indicate the *LlZFHD4* transgenic plants are more tolerant to cold and water stresses than WT plants.

**Figure 8:**
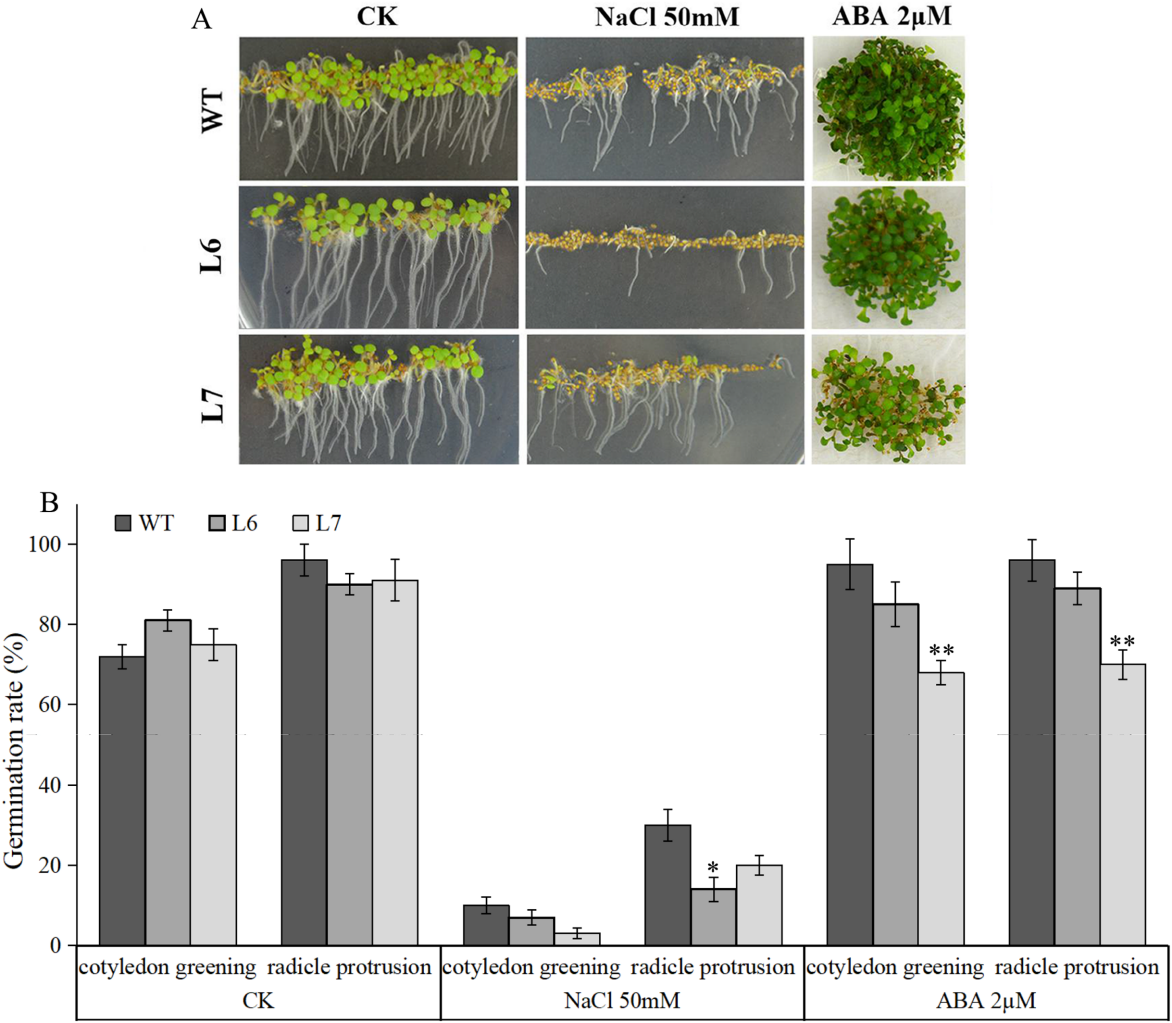
Overexpression of *LlZFHD4* in *Arabidopsis* reduced salinity and ABA tolerance. (A) Observation and (B) seed germination counts of T3 generation *LlZFHD4* transgenic Arabidopsis lines (L6, L7) and WT seeds on MS medium supplemented with 2 µM ABA or 50 mM NaCl after 7 d of incubation at 22 °C. The bars show the mean ±SD of three biological replicates. Significant differences between the L6, L7 and WT plants are indicated as 0.01 < *p-value < 0.05 and **p-value < 0.01.

Under salt treatment, however, lower germination ratios measured by radicle protrusion rate were observed in L6 and L7, especially in L7, than in WT on the MS agar plates supplemented with 50 mM NaCl (Figs. 8A, 8B). We also found L6 and L7, especially L6, displayed lower germination ratios than WT under ABA (2 µM) treatment measured by both radicle protrusion and cotyledon greening (Figs. 8A, 8B). These results indicate the *LlZFHD4* transgenic plants might be less tolerant to salinity and more hypersensitive to ABA than WT plants.

### Overexpressing *LlZFHD4* increases the expression of stress- and ABA-responsive genes

**Figure 9:**
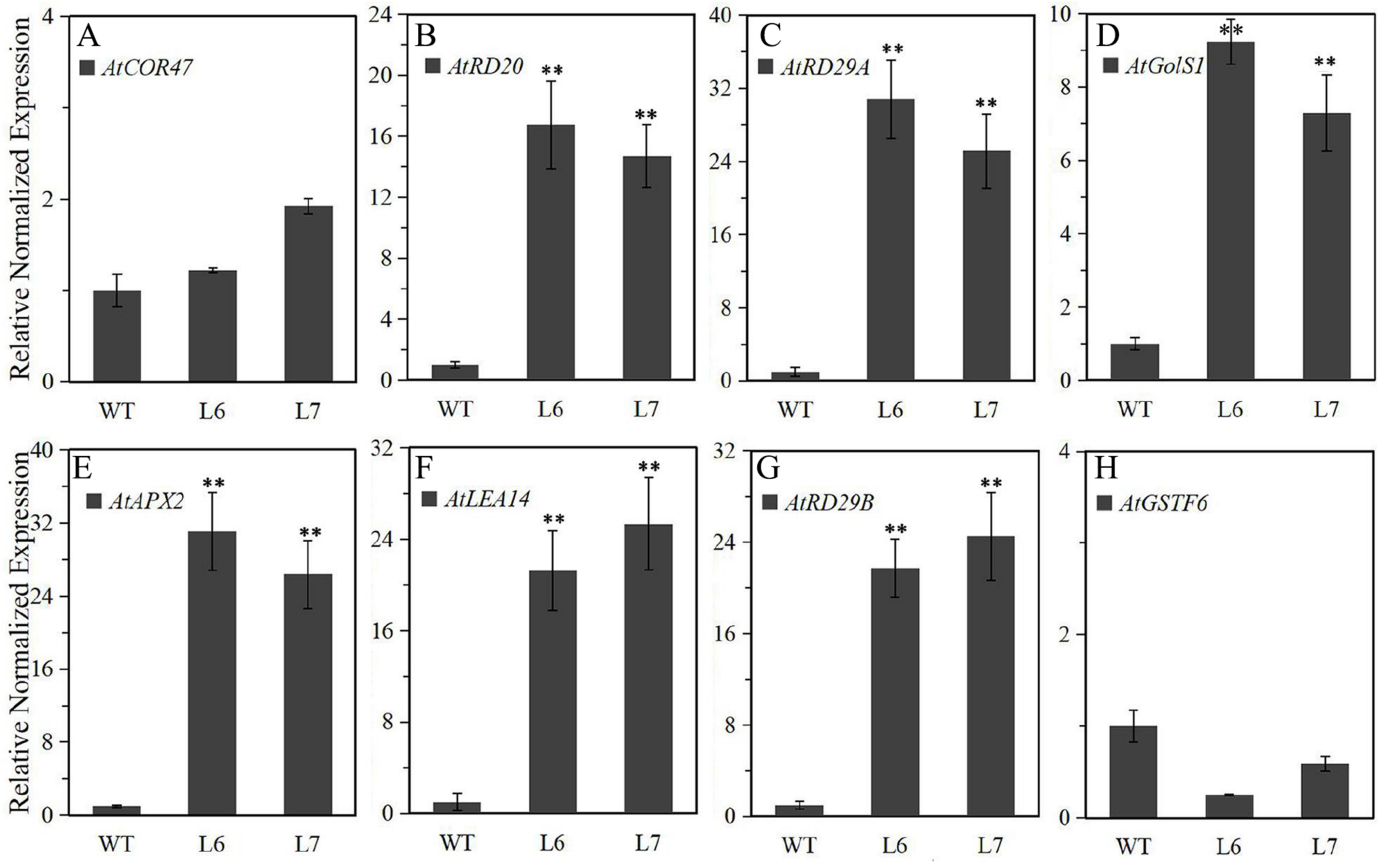
Expression level of the stress-responsive genes in *LlZFHD4* transgenic *Arabidopsis* under normal conditions. qRT-PCR was performed on the *Arabidopsis* (A) *AtCOR47*, (B) *AtRD20*, (C) *AtRD29A*, (D) *AtGolS1*, (E) *AtAPX2*, (F) *AtLEA14*, (G) *AtRD29B* and (H) *AtGSTF6* genes. The reference gene *Atactin* (NM_112764) was used as an internal reference gene. The expression level of WT samples was set as 1. Significant differences between the T3 generation *LlZFHD4* transgenic Arabidopsis lines (L6, L7) and WT seedlings are indicated as **p-value < 0.01.

To further explore the molecular mechanism underlying *LlZFHD4* in response to osmotic stresses, we assessed the expression levels of some known stress- and ABA-responsive genes in *LlZFHD4* transgenic *Arabidopsis* under normal growth conditions. The results showed that the transcripts of *AtRD29A*, *AtRD20*, *AtGolS1*, *AtLEA14*, *AtAPX2*, and *AtRD29B* genes (Table S1) accumulated significantly higher in L6 and L7 than WT seedlings (Fig. 9). More importantly, the expression level of *AtRD29A*, *AtRD29B*, and *AtAPX2* in L6 and L7 was even more than 20 folds of that in WT (Fig. 9), implying LlZFHD4 may confer cold and water stress tolerances by effectively up-regulating stress- and ABA-responsive genes.

## Discussion

In this study, we identified a novel stress-responsive ZFHD TF gene, *LlZFHD4*, from the wild lily species *Lilium lancifolium*. Sequence analysis showed that LlZFHD4 contains conserved domains in both the N-terminal and the C-terminal regions. The N-terminal domain contains five conserved cysteine and two conserved histidine residues, whereas the C-terminus harbors a conversed DNA-binding homeodomain. LlZFHD4 protein is also shown to be a nucleus-localized transcriptional activator with transactivation activity in the N-terminus.

Members of the ZFHD TF family in *Arabidopsis*, wheat, barley, Chinese cabbage, Tartary buckwheat (*Fagopyrum tataricum*), cucumber and tobacco are reported to function as transcriptional regulators in floral development or stress response processes (Abu-Romman, 2014; Abu-Romman & Al-Hadid, 2017; Lai et al., 2021; Khatun et al., 2017; Liu et al., 2019; Sun et al., 2021; Tan & Irish, 2006). In this study, we found that *LlZFHD4* may play similar roles in tiger lily. The qRT-PCR results showed that *LlZFHD4* has the highest expression levels in flower petals. Cold, drought, salt, and ABA treatments can also significantly up-regulate the expression level of *LlZFHD4*. Furthermore, the expression of the *GUS* gene driven by the *LlZFHD4* promoter could be up-regulated by cold, salt, and water stresses, and ABA. Thus, we suppose the *LlZFHD4* promoter has higher transcript activation activity under cold, drought, salt, and ABA conditions. On the other hand, the expression of *LlZFHD4* in response to stresses may also be regulated by some upstream regulatory factors like DREB, MYC, and WRKY TFs for their binding sites (MYC, DRE, and W-box) are located in the *LlZFHD4* promoter region. This may be the reason why the expression patterns of *LlZFHD4* were different from the expression patterns of *GUS* driven by *LlZFHD4* promoter under stress treatments.

Recent studies on *ZFHD* genes in tomato and tobacco has revealed that the silencing of the *SL-ZH13* gene exhibited reduced drought and salt tolerance of transgenic tomato (Zhao et al., 2019), and silencing of *NtZFHD21* decreased the drought tolerance of transgenic tobacco (Sun et al., 2021). Additionally, many researchers have reported that C2H2-type zinc finger proteins play crucial roles, acting both positively and negatively in abiotic stress signaling in *Arabidopsis*. For instance, overexpression of *Zat12* in *Arabidopsis* could not only result in enhanced tolerance of freezing, osmotic, salinity, oxidative and light stresses and iron deficiency (Davletova et al., 2005; Vogel et al., 2005); but also conferred enhanced heat sensitivity in contrast (Le et al., 2016). Similarly, constitutive expression of *Zat10* in transgenic plants was found to improve drought stress tolerance; but suppress defense responses that enhance osmotic and salinity stress tolerance (Mittler et al., 2006). Moreover, AZF2 and STZ function as transcriptional repressors to increase drought, cold, and salinity tolerance by negatively regulating ABA-repressive and auxin-inducible genes (Kodaira et al., 2011; Sakamoto et al., 2004). In this study, we found that *LlZFHD4* plays both positive and negative roles in plant stress responses.

Under cold and water stress conditions, compared to WT seedlings, better growth status, higher survival rate, higher soluble sugar level, lower electrolyte leakage amount, and lower leaf water-loss ratios were observed in *LlZFHD4* transgenic lines. Meanwhile, the expression levels of 6 well-known stress-related genes from *Arabidopsis* (*AtRD29A*, *AtRD20*, *AtGolS1*, *AtLEA14*, *AtAPX2*, and *AtRD29B*) were significantly higher (approximately ten to thirtyfold) in *LlZFHD4* transgenic lines than in WT. Therefore, we assume LlZFHD4 TF can directly or indirectly regulate these genes at the transcriptional level to activate the plant adaptation to cold and drought stresses. In contrast, overexpression of *LlZFHD4* had resulted in reduced salt tolerance in *Arabidopsis*, suggesting the LlZFHD4 may play a negative role in the plant adaptive response to salt stress. However, the molecular mechanism underlying this phenotype needs to be further illustrated.

On the other hand, some *ZFHD* genes from *Arabidopsis*, rice, and Chinese cabbage have been reported to involve in ABA signaling pathway responding to stresses (Tran et al., 2007; Figueiredo et al., 2012; Wang et al., 2016). In this study, the expression level and promoter activity of *LlZFHD4* can be induced by ABA treatment; the *LlZFHD4* transgenic *Arabidopsis* showed enhanced ABA sensitivity than WT plants. Considering the induced stress-related genes in *LlZFHD4* transgenic lines are also known to be ABA-responsive genes, these results suggest *LlZFHD4* may somewhat rely on ABA signaling to function in plant adaptation to abiotic stresses.

## Conclusions

*LlZFHD4* is a stress-responsive gene induced under cold, drought, salt, and ABA conditions. The expression of *LlZFHD4* responding to osmotic stresses may be activated by the stress-inducible promoter; and, or regulated by the upstream regulatory factors like DREB, MYC, and WRKY TFs, for their binding sites are located in the *LlZFHD4* promoter region. Overexpression of *LlZFHD4* can up-regulate the expression of some stress- and ABA-responsive functional genes in *Arabidopsis*; thus, under freezing and drought conditions, *LlZFHD4* transgenic *Arabidopsis* showed better growth status, higher survival rates, and higher osmotic adjustment capacity than WT. Meanwhile, reduced salt and ABA tolerance were observed in *LlZFHD4* transgenic *Arabidopsis*. Our findings provide a novel *ZFHD* gene that may play positive role in cold and drought stress response, whereas function negatively in salinity tolerance, through the ABA signaling pathway.

## Supplemental Information

10.7717/peerj.11508/supp-1 Supplemental Information 1 The CDS and amino acid sequence of *LlZFHD4*.

10.7717/peerj.11508/supp-2 Supplemental Information 2 The promoter sequence of *LlZFHD4*.

10.7717/peerj.11508/supp-3 Supplemental Information 3 Overexpression of *LlZFHD4* confirmed by qRT-PCR. 12 independent T2-generation transgenic plants were chosen for the analysis. The line 6 and 7 (L6 and L7) which showed relative high transcript levels of *LlZFHD4* were chosen for further study.

10.7717/peerj.11508/supp-4 Supplemental Information 4 Primers used in qRT-PCR analysis and LlZFHD4 transcription activation assay.

10.7717/peerj.11508/supp-5 Supplemental Information 5 The raw data of expression patterns of *LlZFHD4* in tissues and under abiotic stresses. Relative expression levels of *LlZFHD4* normalized against levels of *LlTIP1* as calculated by qRT-PCR (mean ±SD of three biological replicates).

10.7717/peerj.11508/supp-6 Supplemental Information 6 The raw data of expression patterns of *GUS* gene driven by *LlZFHD4* promoter under abiotic stresses. Relative expression levels of *GUS* normalized against levels of *Atactin* as calculated by qRT-PCR (mean ±SD of three biological replicates).

10.7717/peerj.11508/supp-7 Supplemental Information 7 The raw data of relative electrolyte leakage and soluble sugar content in WT and transgenic Arabidopsis (L6, L7) after 4 °C and 16.1% PEG treatments; and the rate of water-loss from WT and L6, L7 cutting leaves.

10.7717/peerj.11508/supp-8 Supplemental Information 8 The raw data of expression levels of stress-related genes in WT and *LlZFHD4* overexpressing lines under normal condition. qRT-PCR was performed on the *Arabidopsis AtRD29A*, *AtRD20*, *AtGolS1*, *AtLEA14*, *AtAPX2* and *AtRD29B* genes. The reference gene *Atactin* (NM_112764) was used as an internal reference gene. The expression level of WT samples was set as 1.

## Additional Information and Declarations

### Data Availability

The following information was supplied regarding data availability: Raw measurements are available in the Supplemental Figures and Tables.
