# Supplementary material for: Functional characterization of Lilium lancifolium cold-responsive Zinc Finger Homeodomain (ZFHD) gene in abscisic acid and osmotic stress tolerance
Source: PeerJ. 2021 May 25;9:e11508. doi: 10.7717/peerj.11508 (PMC8162235; doi:10.7717/peerj.11508)
Supplement: Supplemental Information 1 [file peerj-09-11508-s001.pdf]

1 ATGGATCTCCCCATTTATCAAGGGGATATCCATGACAACCACAAC
M D L P I Y Q G D I H D N H N
46 AAAAGTGGGGCGGCGGCGGCGGTGAAGTACAAGGAGTGCCTGAAG
K S G A A A A V K Y K E C L K
91 AACCATGCAGCCACCATTGGAGTCATTGCCATTGATGGGTGTGGG
N H A A T I G V I A I D G C G
136 GAGTTCATGCCGAGTGGGGAGGAGGGCACACTGGAGGCCCTTAGA
E F M P S G E E G T L E A L R
181 TGCTCAGCCTGCGGCTGCCACCGGAACTTCCACAGAAAAGAGACT
C S A C G C H R N F H R K E T
226 GAAGGAGAGGCTTCATCGTGTGGGTGCTCCCACAAGCTCTTGTCA
E G E A S S C G C S H K L L S
271 CCGCACCATATGATCATGCCCTTGGCCCTGCAGGCCTCGGAGCCA
P H H M I M P L A L Q A S E P
316 TTTGGCATGGTGAGGAAGAGATTCAGGACCAAGTTCACGCCGGGG
F G M V R K R F R T K F T P G
361 CAGAAGGAGAAGATGCTAGGGTTTGCTGAAAGAGTTGGATGGAAG
Q K E K M L G F A E R V G W K
406 ATGCAGAGGCTGGATGAGGGTGTGGTGCAGCAGTTCTGTCAGGAG
M Q R L D E G V V Q Q F C Q E
451 GTTGGGGTGAAGAGGAGGGTGCTCAAGGTTTGGATGCACAACAAC
V G V K R R V L K V W M H N N
496 AAGCAGAATTTGGCAAAAAAGGATCCATTGCAGAGTGACTACTGA
K Q N L A K K D P L Q S D Y *
