# Supplementary material for: Functional characterization of Lilium lancifolium cold-responsive Zinc Finger Homeodomain (ZFHD) gene in abscisic acid and osmotic stress tolerance
Source: PeerJ. 2021 May 25;9:e11508. doi: 10.7717/peerj.11508 (PMC8162235; doi:10.7717/peerj.11508)
Supplement: Supplemental Information 2 [file peerj-09-11508-s002.pdf]

-781 GCTTGATATC GAATTCGCGT GTCGCCCTTA CTATAGGGCA CGCGTGGTCG ACGGCCCGGG

-721 CTGGTATTGA GCCATTTGTT TAATTATTCA AGATTTACAA CAACAACAAA AATTAAATCC

-661 TTTAACTTAT GAAACACTTT AAATTTCTCT GTTCATTAAT CCAGCCAAGA CCATTCCCTT

-601 ATCTCCCTTT CTCTGCCCAC TATGGTGTCC CCTCTCTACT GCCATAGCCT ATAAGCTTCT

-541 TATTAAGGGG CTCACCAACA CAACCATGCA CCCCAAATGA CTCAAATTCA AGCCCTTCTC

-481 TCTCTCAGTT TCATCTTCTT CCCATTGTAA CCACCTTTTA AACCTTCATC CTGCCATGAA

-421 ATGCAACACT GAAGAATAGT TAAACTTTCC ATTTTCCCCC TGCACTTCCT TCATCCCTCG

-361 GCACTCCTCG AAGAGACCTA ATTACTGTTT GACCCTAGCA GCCACCCCCA CCATTCTCTT

-301 TCTATCCCTC TCTAACAACC TAAGACGGAT GTGAAATTGG CTTTATTGTG TTGTTCTCTG

-241 TTGGTTTTCT CTCCTCACCT AAGATAAGGT TATCTAGTTC CTTAGAGGTT CCAAATTTTT

-181 CCACAGCCTC AAAGCAAAAC AAAAATCTCG CAAGTGGCTT GGTTGTTGTT TTATTTAGTG

-121 TCCAAAAAAA TCTGACCCAA ATTCCATTCG ATAATTTCCT GTAGTTTGCG TGGTCTAATT

-61 CTCTCCATTT TCCCTTTTTC CTTTTAGTCT CCAATAAAAT AAAAAGGATC CTAAATTGAG

-1 A<u>ATG</u>
