# Supplementary figures and images for: Functional characterization of Lilium lancifolium cold-responsive Zinc Finger Homeodomain (ZFHD) gene in abscisic acid and osmotic stress tolerance
Source: PeerJ. 2021 May 25;9:e11508. doi: 10.7717/peerj.11508 (PMC8162235; doi:10.7717/peerj.11508)

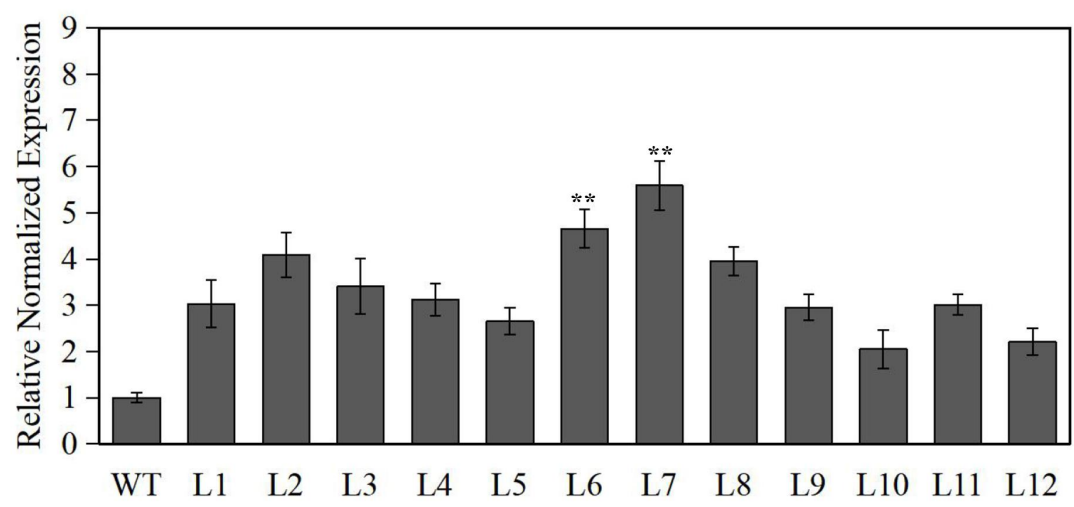

Supplement: Supplemental Information 3 — 12 independent T2-generation transgenic plants were chosen for the analysis. The line 6 and 7 (L6 and L7) which showed relative high transcript levels of LlZFHD4 were chosen for further study. [file peerj-09-11508-s003.pdf]
