# Supplementary material for: Functional characterization of Lilium lancifolium cold-responsive Zinc Finger Homeodomain (ZFHD) gene in abscisic acid and osmotic stress tolerance
Source: PeerJ. 2021 May 25;9:e11508. doi: 10.7717/peerj.11508 (PMC8162235; doi:10.7717/peerj.11508)
Supplement: Supplemental Information 4 [file peerj-09-11508-s004.docx]

| **Primers name** | **Accession** | **Oligonucleotides (5’- 3’)** |
| --- | --- | --- |
| *LlZFHD4* |  | F: GTTTGCTGAAAGAGTTG |
|  |  | R: TTCTGCTTGTTGTTGTG |
| *LlTIP1* |  | F: CGAAGCCAGAAACGGAGAAGAAT |
|  |  | R: CGAAGCCAGAAACGGAGAAGAAT |
| *Atactin* | NM_112764 | F: CTCATGCCATCCTCCGTCTT |
|  |  | R: ACTTGCCCATCGGGTAATTC |
| *AtRD29A* | NM_124610 | F: GAGCAACGAGGGGAAGATAAAAG |
|  |  | R: TCAGTCGCACCACCACCGAACCA |
| *AtRD29B* | NM_124609 | F: CAAAACCAAGCACCTACACA |
|  |  | R: CTCCTTCACTCCACTTCCAC |
| *AtRD20* | NM_128898 | F: ATTCGAGCACCTATGACACC |
|  |  | R: AAACTTCCATCAAAGCAACC |
| *AtCOR47* | NM_101894 | F: TCCCAGGACACCACGACAAGAC |
|  |  | R: CCTCTTCAGTGGTCTTGGCATG |
| *At**APX2* | At3G09640 | F: TCAGGATTCGAGGGTGCATG |
|  |  | R: AAGGCATCCTCATCTGCAGC |
| *At**LEA14* | At1G01470 | F: ACCGGATTTAATTCATTAAGCGCT |
|  |  | R: TCCCAAGCTGGCAGAGGGAAT |
| *AtGolS1* | At2g47180 | F: AGCCGTTCATCACCGCTCTTAC |
|  |  | R: ACTCCTGGCAACATTCAAGCAG |
| *AtGSTF6* | NM_100174 | F: AAATTGCCGAAATCGAACAG |
|  |  | R: TTTTCCCTCGTTCTGTCTCC |
| *GUS* | AHB37685.1 | F: GGGCAACAAGCCGAAAGA |
|  |  | R: GCCAGTGGCGCGAAATAT |
| pGBKT7-LlZFHD4-A |  | F: CATGGAGGCCGAATTC(*EcoR*I)ATGGATCTCCCCATTTATC |
|  |  | R: GCAGGTCGACGGATCC(*BamH*I)TCAGTAGTCACTCTGCAATG |
| pGBKT7-LlZFHD4-N |  | F: CATGGAGGCCGAATTC(*EcoR*I)ATGGATCTCCCCATTTATC |
|  |  | R: GCAGGTCGACGGATCC(*BamH*I)TGACAAGAGCTTGTGGGAG |
| pGBKT7-LlZFHD4-C |  | F: CATGGAGGCCGAATTC(*EcoR*I)CCGCACCATATGATCATGC |
|  |  | R: GCAGGTCGACGGATCC(*BamH*I) TCAGTAGTCACTCTGCAATG |
